# Immune-escape mutations and stop-codons in HBsAg develop in a large proportion of patients with chronic HBV infection exposed to anti-HBV drugs in Europe

**DOI:** 10.1186/s12879-018-3161-2

**Published:** 2018-06-01

**Authors:** Luna Colagrossi, Lucas E. Hermans, Romina Salpini, Domenico Di Carlo, Suzan D. Pas, Marta Alvarez, Ziv Ben-Ari, Greet Boland, Bianca Bruzzone, Nicola Coppola, Carole Seguin-Devaux, Tomasz Dyda, Federico Garcia, Rolf Kaiser, Sukran Köse, Henrik Krarup, Ivana Lazarevic, Maja M. Lunar, Sarah Maylin, Valeria Micheli, Orna Mor, Simona Paraschiv, Dimitros Paraskevis, Mario Poljak, Elisabeth Puchhammer-Stöckl, François Simon, Maja Stanojevic, Kathrine Stene-Johansen, Nijaz Tihic, Pascale Trimoulet, Jens Verheyen, Adriana Vince, Snjezana Zidovec Lepej, Nina Weis, Tülay Yalcinkaya, Charles A. B. Boucher, Annemarie M. J. Wensing, Carlo F. Perno, Valentina Svicher, on behalf of the HEPVIR working group of the European Society for translational antiviral research (ESAR)

**Affiliations:** 1 Department of Experimental Medicine and Surgery, University of Rome Tor Vergata, Via Montpellier, 1, 00133 Rome, Italy; 2 Virology, Department of Medical Microbiology, University Medical Centre Utrecht, Utrecht, The Netherlands; 3 Department of Viroscience, Erasmus Medical Centre, Rotterdam, The Netherlands; 4 Servicio de Microbiología, Hospital San Cecilio, Instituto de Investigación Biosanitaria ibs.GRANADA, Hospitales Universitarios de Granada, Granada, Spain; 5 Hygiene Unit, IRCCS AOU San Martino – IST, Genoa, Italy; 6 Malattie Infettive, Seconda Università degli studi di Napoli, Naples, Italy; 7 Laboratory of Retrovirology, CRP-Santé, Luxembourg, Luxembourg; 8 Molecular Diagnostics Laboratory, Hospital of Infectious Diseases, Warsaw, Poland; 9 Institute of Virology, University of Cologne, Cologne, Germany; 10 Izmir Tepecik Education and Research Hospital, Clinic of Infectious Diseases and Clinical Microbiology, Izmir, Turkey; 11 Section of Molecular Diagnostics, Clinical Biochemistry, Aalborg University Hospital, Aalborg, Denmark; 12 Institute of Microbiology and Immunology, Faculty of Medicine, University of Belgrade, Belgrade, Serbia; 13 Institute of Microbiology and Immunology, Faculty of Medicine, University of Ljubljana, Ljubljana, Slovenia; 14 Service de Microbiologie, University Paris Diderot, Hôpital Saint Louis, Paris, France; 15 L. Sacco Hospital, Milan, Italy; 16 National HIV Reference Laboratory, Central Virology Laboratory, Ministry of Health, Tel Hashomer, Ramat Gan, Israel; 17 Molecular Diagnostics Laboratory, National Institute for Infectious Diseases “Matei Bals”, Bucharest, Romania; 18 National Retrovirus Reference Centre, Department of Hygiene, Epidemiology and Medical Statistics, Faculty of Medicine, National and Kapodistrian University of Athens, Athens, Greece; 19 Department for Virology, Medical University of Vienna, Vienna, Austria; 20 Liver Disease Centre, Sheba Medical Centre, Ramat Gan, Israel; 21 Department of Virology, Norwegian Institute of Public Health, Oslo, Norway; 22 Institute of Microbiology, Polyclinic for Laboratory Diagnostics, University Clinical Centre Tuzla, Tuzla, Bosnia and Herzegovina; 23 Virology Laboratory, Centre Hospitalier Régional et Université “Victor Segalen”, Bordeaux, France; 24 Institute of Virology, University-Hospital, University Duisburg-Essen, Essen, Germany; 25 University of Zagreb School of Medicine and University Hospital for Infectious Diseases, Zagreb, Croatia; 26 Department of Infectious Diseases, Copenhagen University Hospital, Hvidovre, Copenhagen, Denmark; 27 Refik Saydam National Public Health Agency, Ankara, Turkey

**Keywords:** HBV, HBsAg, Immune-escape, Stop-codons, Drug-resistance

## Abstract

**Background:**

HBsAg immune-escape mutations can favor HBV-transmission also in vaccinated individuals, promote immunosuppression-driven HBV-reactivation, and increase fitness of drug-resistant strains. Stop-codons can enhance HBV oncogenic-properties. Furthermore, as a consequence of the overlapping structure of HBV genome, some immune-escape mutations or stop-codons in HBsAg can derive from drug-resistance mutations in RT. This study is aimed at gaining insight in prevalence and characteristics of immune-associated escape mutations, and stop-codons in HBsAg in chronically HBV-infected patients experiencing nucleos(t)ide analogues (NA) in Europe.

**Methods:**

This study analyzed 828 chronically HBV-infected European patients exposed to ≥ 1 NA, with detectable HBV-DNA and with an available HBsAg-sequence.

The immune-associated escape mutations and the NA-induced immune-escape mutations sI195M, sI196S, and sE164D (resulting from drug-resistance mutation rtM204 V, rtM204I, and rtV173L) were retrieved from literature and examined. Mutations were defined as an aminoacid substitution with respect to a genotype A or D reference sequence.

**Results:**

At least one immune-associated escape mutation was detected in 22.1% of patients with rising temporal-trend. By multivariable-analysis, genotype-D correlated with higher selection of ≥ 1 immune-associated escape mutation (OR[95%CI]:2.20[1.32–3.67], *P* = 0.002). In genotype-D, the presence of ≥ 1 immune-associated escape mutations was significantly higher in drug-exposed patients with drug-resistant strains than with wild-type virus (29.5% vs 20.3% *P* = 0.012). Result confirmed by analysing drug-naïve patients (29.5% vs 21.2%, *P* = 0.032). Strong correlation was observed between sP120T and rtM204I/V (*P* < 0.001), and their co-presence determined an increased HBV-DNA.

At least one NA-induced immune-escape mutation occurred in 28.6% of patients, and their selection correlated with genotype-A (OR[95%CI]:2.03[1.32–3.10],*P* = 0.001).

Finally, stop-codons are present in 8.4% of patients also at HBsAg-positions 172 and 182, described to enhance viral oncogenic-properties.

**Conclusions:**

Immune-escape mutations and stop-codons develop in a large fraction of NA-exposed patients from Europe. This may represent a potential threat for horizontal and vertical HBV transmission also to vaccinated persons, and fuel drug-resistance emergence.

**Electronic supplementary material:**

The online version of this article (10.1186/s12879-018-3161-2) contains supplementary material, which is available to authorized users.

## Background

Worldwide, around 250 million individuals have a chronic hepatitis B virus (HBV) infection. Among them, around 1 million dies as a consequence of end-stage liver disease or hepatocellular carcinoma (HCC) [1].

HBV is a highly evolving pathogen characterized by a high degree of genetic-variability (a unique property among DNA viruses) that is driven by the lack of proof-reading function of HBV reverse transcriptase (RT) and exacerbated by the high speed of the HBV replication cycle [2].

This high degree of HBV genetic-variability allows the virus to react to endogenous (i.e. immune system), and exogenous (i.e. vaccination, hepatitis B immunoglobulin, antiviral drugs) selective pressures by further modulating its genome structure.

Among the different HBV-proteins, HBV surface antigen (HBsAg) contains the major hydrophilic region that is a dominant epitope crucial for binding to neutralizing-antibodies. So far, around 30 immune-escape mutations in HBsAg (hereafter defined as immune-associated escape mutations), have been identified [3–5] to evade neutralizing-antibodies, to allow persistent HBV-infection and to promote viral fitness [2, 6]. These mutations can have relevant pathobiological implications at the time of immunosuppression-driven HBV-reactivation, thus favoring the reuptake of viral replication during the initial weakening of immune responses [6–9]. Immune-associated escape mutations can also hamper HBsAg-recognition by antibodies induced by vaccine, thus posing a potential threat for the global vaccination program also in the setting of mother-to-child transmission [2]. In addition, Immune-associated escape mutations can decrease/abrogate HBsAg-binding to antibodies used in diagnostic assays for HBsAg-detection and -quantification [6, 10, 11], and thus determine a false-negativity or an underestimation of HBsAg levels, that can pose an issue for a proper diagnosis and staging of chronic HBV-infection.

To date, six nucleos(t)ide analogues (NAs) have been approved for the treatment of HBV-infection, namely lamivudine (LAM), adefovir dipivoxil (ADV), entecavir (ETV), telbivudine (LdT), tenofovir (TDF), and the recently approved tenofovir-alafenamide (TAF). Among them ETV, TDF or TAF are characterized by high genetic barrier to resistance [12], and thus they are preferred as first-line treatment in the majority of European Countries [13–15].

Furthermore, due to the overlapping between the genes encoding reverse transcriptase (RT) and HBsAg, some RT drug-resistance mutations can introduce mutations in the major hydrophilic region of HBsAg that are capable to reduce the binding affinity for neutralizing antibodies, including those induced by HBV-vaccine [16]. Again, these mutations (hereafter defined as NA-induced immune-escape mutations) may pose a public health concern for their pathogenetic potential and possibility of transmission to vaccinated individuals.

Another type of mutation that can be detected in HBsAg is represented by stop-codons. They are associated with the synthesis of truncated forms of HBsAg that remain trapped in the endoplasmic reticulum. This intracellular HBsAg accumulation can induce an oxidative stress that can favour the neoplastic transformation of hepatocytes [17].

Information about the prevalence of the above-mentioned mutations in patients with chronic HBV-infection exposed to NA in Europe is limited. Filling this gap can provide an estimate of the pool for HBV-transmissions also to vaccinated individuals and/or can have a higher risk of disease progression. Thus, this study was designed to estimate the prevalence and characteristics of i) immune-associated escape mutations ii) NA-induced immune-escape mutations and iii) stop-codons in HBsAg in Europe.

## Methods

### Study population

A multicenter survey was performed on genotypic-resistance testing results generated during routine clinical assessments of patients with chronic hepatitis B attending tertiary referral centers in European countries according to Hermans et al., 2016. Inclusion criteria were: chronic hepatitis B with detectable serum HBV-DNA, exposure to ≥ 1 NA, RT/HBsAg-sequence availability, and age ≥ 18 years [18]. Inclusion of patients exposed to NAs allows to define the prevalence of immune-associated, and also of NA-induced escape mutations.

935 RT/HBsAg-sequences were collected in the time-window between January 1998 and August 2012. Only 1 sequence per patient was included in the analysis. Patient datasets were collected in the framework of the European Society for translational antiviral research (ESAR) from 15 countries. Countries were grouped in geographical regions (http://unstats.un.org/unsd/) as follows: Northern Europe (Denmark/Norway), Western Europe (Austria/France/Germany/Luxembourg/Netherlands), Eastern Europe (Poland/Romania), and Southern Europe (Greece/Italy/Serbia/Slovenia/Spain) [19]. Israel and Turkey were grouped with Southern European countries [18].

### Data characteristics

The following information was collected: serum HBV-DNA; HBsAg; hepatitis B e antigen (HBeAg); anti-HBe; serum–alanine aminotransferase (serum-ALT); exposure to ≥ 1 NA (LAM, LdT, ADV, ETV, TDF, LdT). No administrative permissions were required to review patients’ records and to use related data.

### RT/HBsAg sequencing

RT/HBsAg sequences obtained by well-standardized population-based sequencing procedures during routine clinical practise were collected. Sequence data consisted of FASTA files containing nucleic acid sequence information of the RT/HBsAg region. The ESAR quality control procedure was applied on all submitted sequences. If amino acid substitutions at immune-escape codons were due to ambiguities consisting of > 2 bases per nucleotide position or > 1 ambiguities per codon, or if insertions or deletions were present causing a shift in the HBsAg open-reading frame that affected immune-escape codons, sequences were excluded from the analysis [18]. Furthermore, there was no specific pattern of mutations linked to a specific center.

HBsAg sequences were analyzed using SeqScape-v2.6 software (Thermo-Fisher Scientific), then the sequences were aligned using Bioedit 7.0 software [20]. Sequences having a mixture of wild-type and mutant residues at single positions were considered to have the mutant(s) at that position. The mixed base identification was set at a percentage of 20%.

HBsAg sequences have been submitted to Genbank with the following accession number: MH218870-MH219804.

### Mutation prevalence

HBsAg-sequences were analysed to define the prevalence of immune-associated escape mutations, NA-induced escape mutations, and stop-codons.

Mutations were defined as difference from HBV genotype-A reference sequence (Genbank accession number: **JN182318**) or HBV genotype-D reference sequence (Genbank accession number: **GU456636**).

We determined the prevalence of 29 immune-associated escape mutations (sQ101K, sT114R, sP120S/T/A, sT123A/N, sT126N/S, sP127L, sA128V, sQ129R/N, sG130N/R, sT131I, sM133I/L/T, sY134L, sC138Y, sC139S, sT140S, sP142S, sD144A/E, sG145A/R, sN146S) extensively retrieved from literature and known to affect HBsAg-recognition by antibodies [3–5, 19]. Among them, sP120S/T/A, sT126N/S, sQ129R/N, sT131I/N, sM133I/L, sP142S, sD144A/E, sG145A/R were known to act as vaccine-escape mutations [3–5, 19]. All these mutations are localized in the major hydrophilic region of HBsAg known to contain the major B-cell epitopes.

We also analyzed the prevalence of the NA-induced immune-escape mutations sI195M, sI196S, and sE164D (resulting from drug-resistance mutation rtM204 V, rtM204I, and rtV173L) [12] and stop-codons.

### Statistical analysis

Statistical analysis was performed using SPSS software (v19.0; SPSS Inc., Chicago, IL) and the statistical environment R (version 3.2.5). Data were expressed as median (interquartile range [IQR]) for quantitative variables and as counts and percentages for qualitative variables. Chi-Squared Test of Independence based on a 2 × 2 contingency table was used for qualitative data, while Mann-Whitney test for continuous data.

Univariable and multivariable logistic regression analysis was performed in order to assess the potential associations between the presence of at least one i) immune-associated escape mutation, ii) NA-induced immune-escape mutation, iii) stop-codon, with several factors, including: gender, age, serum HBV-DNA at the time of genotypic testing, LAM, ADV, ETV, TDF, geographical origin, year of collection, and HBV-genotype.

## Results

### Study population

The study population included 935 patients with chronic HBV infection exposed to ≥ 1 NA. Phylogenetic analysis showed that most patients were infected with HBV genotype-D (573, 61.3%) and genotype-A (255, 27.3%). In the remaining patients, the following HBV-genotypes were detected: B (36, 3.9%), C (36, 3.9%), E (23, 2.4%), G (5, 0.5%), H (4, 0.4%), F (3, 0.3%).

To provide a more robust characterization of immune-escape mutations and stop-codons circulating in Europe, the analysis was focused on 828 patients infected with HBV genotype-D and A. Table 1 shows demographics, clinical, biochemical, and virological characteristics of these patients.

**Table 1:** Patients’ Characteristics

	Overall (*N* = 828)	Genotype-A (*N* = 255)	Genotype-D (*N* = 573)	*P*-value^d^
General				
Median Age (IQR), years	45 (38–59)	45 (33–56)	49 (40–59)	*0.001*
Male, N(%)^a^	584 (70.5)	183 (74.4)	401 (73.6)	0.810
CHB-related data				
Median HBV-DNA, log IU/ml (IQR)	4.4 (3.2–6.4)	4.7(3.3–6.9)	4.4 (3.2–6.3)	0.079
HBeAg positive, N(%)^b^	183 (44.1)	71 (59.7)	112 (38)	*< 0.001*
Median ALT, IU/L (IQR)	46.5 (32–78)	46 (30–80)	48 (32–78)	0.473
Geographical origin, N(%)				
Western Europe	142 (17.1)	67 (26.3)	75 (13.1)	*< 0.001*
Northern Europe	26 (3.1)	10 (3.9)	16 (2.8)	0.519
Eastern Europe	131 (15.8)	99 (38.8)	32 (5.6)	*< 0.001*
Southern Europe	529 (63.9)	79 (31)	450 (78.5)	*< 0.001*
Anti-HBV drug history, N(%)^c^				
*Monotherapy*				
LAM	406 (62.5)	157 (66.8)	249 (60)	0.085
ADV	32 (4.9)	10 (4.3)	22 (5.3)	0.554
ETV	31 (4.8)	7 (3)	24 (5.8)	0.107
TDF	5 (0.8)	2 (0.9)	3 (0.7)	0.857
LdT	3 (0.5)	1 (0.4)	2 (0.5)	1.000
*Dual exposure*				
LAM + ADV	115 (17.7)	27 (11.5)	88 (21.2)	*0.002*
LAM + TDF	21 (3.2)	14 (6)	7 (1.7)	*0.003*
LAM + ETV	17 (2.6)	10 (4.3)	7 (1.7)	*0.045*
ADV + ETV	4 (0.6)	3 (1.3)	1 (0.2)	0.137
ETV + TDF	3 (0.5)	3 (1.3)	0 (0)	*0.047*
ADV + ETV	1 (0.2)	0 (0)	1 (0.2)	1.000
*Triple exposure*				
LAM + ADV + ETV	5 (0.8)	1 (0.4)	4 (1)	0.450
LAM + ADV + TDF	7 (1.1)	0 (0)	7 (1.7)	*0.045*

^a^ Percentages are calculated on 791 patients with the datum available, 246 patients for genotype A and 545 for genotype D

^b^ Percentages are calculated on 414 patients with the datum available, 119 patients for genotype A and 295 for genotype D

^c^ Percentages are calculated on 650 patients with the type of anti-HBV drugs available, 235 patients for genotype A and 415 for genotype D

^d^ Statistically significant difference was assessed by Chi-squared Test based on a 2 × 2 contingency table

*P*-value in italic are statistically significant

*Abbreviations*: *ADV* adefovir, *ETV* entecavir, *IQR* interquartile range, *LAM* lamivudine, *LdT* telbivudine, *TDF* tenofovir

Patients were predominantly males (70.5%) with a median (IQR) age of 45(38–59)years (Table 1). Median (IQR) log serum HBV-DNA was 4.4(3.2–6.4)IU/ml, and median (IQR) ALT was 47(32–78)U/L (Table 1). Information on HIV-1 coinfection was known for 445 patients. Among them, 103 patients were HIV co-infected.

### Treatment history and drug resistance

A detailed information of anti-HBV drugs used was available for 650 patients. Most patients were exposed to NA mono-therapy, predominantly with LAM (62.5%, 406/650) followed by ADV (4.9%, 32/650), ETV (4.8%, 31/650), TDF (0.8%, 5/650) and LdT (0.5%, 3/650) (Table 1). Exposure to 2 NAs, either simultaneously or consecutively, most frequently concerned LAM + ADV (17.7%, 115/650), followed by LAM + TDF (3.2%, 21/650), LAM + ETV (2.6%, 17/650), ADV + ETV (0.6%, 4/650), ETV + TDF (0.5%, 3/650) and ADV + TDF (0.2%, 1/650) (Table 1). Triple exposure was present in 1.8% (12/650) of patients.

At least one drug-resistance mutation was detected in 54% (447/828) of patients. In particular, the primary mutation rtM204V (conferring full-resistance to LAM, LdT, and partially to ETV) was observed in 25.8% (214/828) of patients, while rtM204I (conferring full-resistance to LAM and LdT) in 20% (166/828). Conversely, rtA181T and rtA181V (conferring full-resistance to ADV and associated with TDF suboptimal response) were detected in 2.3% (19/828) and 3.6% (30/828) of patients, respectively.

### Detection of immune-associated escape mutations

**Fig. 1:**
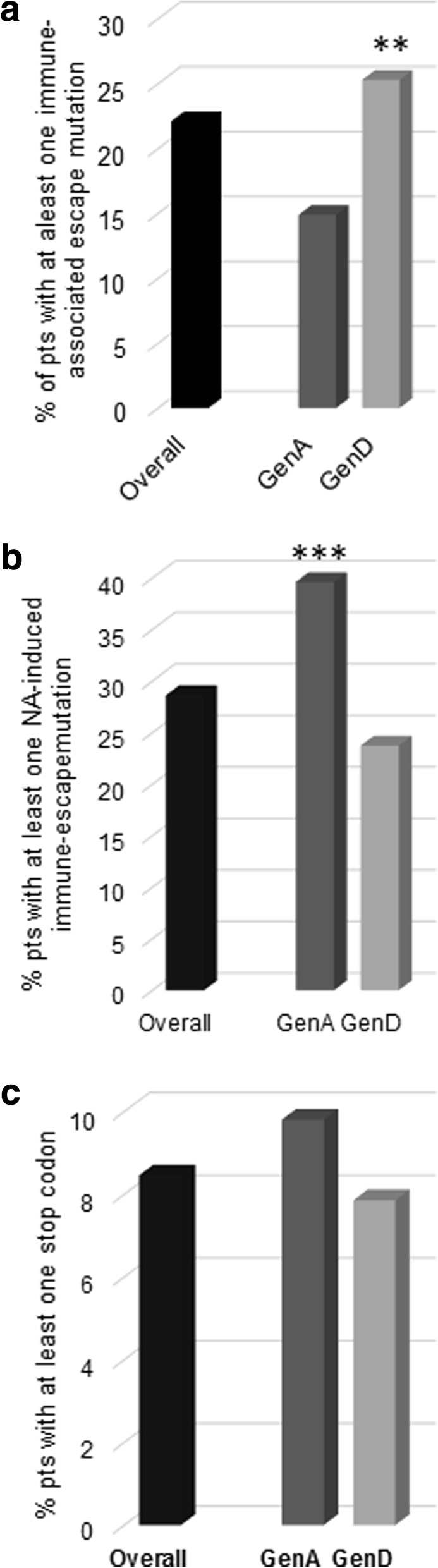
The histograms report the percentage of patients with at least one: **a** immune-associated escape mutation; **b** NA-induced immune-escape mutation; **c** stop-codon. The analyses included a total of 828 chronically HBV-infected patients: 573 infected with HBV genotype-D and 255 with HBV genotype-A. Statistically significant differences were assessed by Chi Square Test based on a 2 × 2 contingency table. **: 0.001; ***: *P* < 0.001. Immune-associated escape mutations (sQ101K, sT114R, sP120S/T/A, sT123A/N, sT126N/S, sP127L, sA128V, sQ129R/N, sG130N/R, sT131I, sM133I/L/T, sY134L, sC138Y, sC139S, sT140S, sP142S, sD144A/E, sG145A/R, sN146S) were retrieved from literature and known to affect HBsAg recognition by antibodies [2, 13, 14, 39–47]. The NA-induced immune-escape mutations I195M, I196S, and E164D result from drug-resistance mutation M204 V, M204I, and V173 L (Torresi, 2002)

At least one immune-associated escape mutation was detected in 22.1% (183/828) of patients (min-max:1–4). In 6% (50/828) of patients, ≥ 2 mutations were detected (Fig. 1a).

The proportion of patients with ≥ 1 immune-associated escape mutation was stable to around 15% (11/73) in 1998–2002 and in 2003–2005 (15/101), showed an increase to 27.2% (89/327) in 2006–2008 (*P* = 0.012, using 1998–2002 as reference), and then declined to 20.8% (68/327) in 2009–2012.

Furthermore, the circulation of HBV strains with ≥ 1 immune-associated escape mutation was significantly higher in genotype-D than A (25.3%[145/573] vs 14.9%[38/255], *P* = 0.001) (Fig. 1a). This result was also observed when the analysis was specifically focused on vaccine-escape mutations (18.3%[105/573] for genotype-D vs 7.1%[18/255] for genotype-A; *P* < 0.001).

**Fig. 2:**
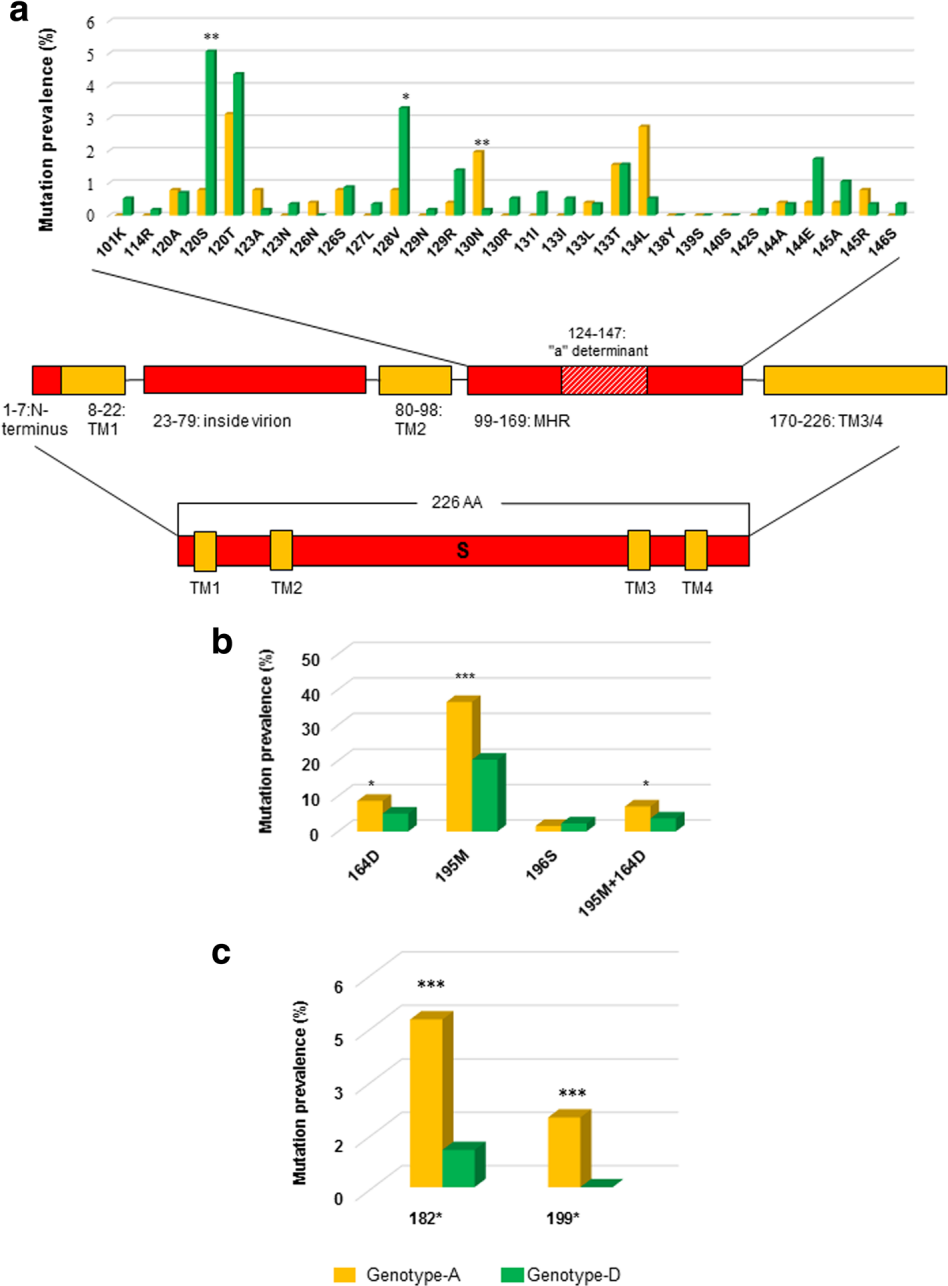
The histograms report the prevalence of **a** immune-associated escape mutations, **b** NA-induced immune-escape mutations, **c** stop-codons. The prevalence was calculated in the group of 255 patients infected with HBV genotype-A (yellow bars) and in the group of 573 patients infected with HBV genotype-D (green bars). Statistically significant differences were assessed by Chi Squared Test for independence based on a 2 × 2 contingency table. * *P* < 0.05; ** *P* < 0.01; *** P < 0.001. In A) a schematic representation of HBsAg functional domains is also reported: N-terminus HBsAg (encompassing amino acids [aa] 1–7), transmembrane domain 1 (TM1, aa: 8–22), loop protruding inside the virion (23-79aa), transmembrane domain 2 (TM2, aa: 80–98), major hydrophilic region (MHR, aa: 99–169) and transmembrane domain 3 and 4 (TM3/4, aa: 170–226). The MHR contains B cell-epitopes including the a-determinant (aa: 124–147)

HBV genotype-D was significantly associated with the selection of specific immune-associated escape mutations. This is the case of sA128V and sP120S selected with higher prevalence in genotype-D than A (sA128V: 3.3%[19/573] vs 0.8%[2/255], *P* = 0.032; sP120S: 5.1%[29/573] vs 0.8[2/255], *P* = 0.003) (Fig. 2a). Conversely, the immune-associated escape mutation G130 N occurred more frequently in genotype-A than D (2%[5/255] vs 0.2%[1/573], P = 0.012) (Fig. 2a). These results were confirmed also when the analysis was focused on LAM-treated patients, thus limiting the impact of anti-HBV drugs on the selection of these mutations (sA128V: 4.4%[16/362] vs 0.5%[2/209], *P* = 0.008; sP120S: 5.5%[20/362] vs 1%[2/209], *P* = 0.006; sG130N: 0.3%[1/362] vs 1.9%[4/209], *P* = 0.063). This suggests that the genetic-backbone of genotype-A and -D can favour the selection of specific immune-associated escape mutations.

In addition, in genotype-D, the presence of ≥ 1 immune-associated escape mutation was significantly higher in drug-exposed patients with drug-resistance than in patients without the drug resistance mutations (29.5%[92/312] vs 20.3%[53/261], P = 0.012). In particular, sP120T significantly correlated with rtM204V/I (P = 0.001): 16/20 patients with sP120T had also rtM204V/I. Moreover, patients with rtM204V/I + sP120T had higher serum HBV-DNA than patients with rtM204V/I alone (5.5[3.2–7.2]logIU/ml vs 4.3[3.2–6.3]logIU/ml). This association was not observed in genotype A.

To corroborate the correlation between immune-associated escape mutations and drug-resistance mutations, the prevalence of ≥ 1 immune-associated escape mutations was also analysed in an independent dataset of drug-naïve patients (cite Additional file 1: Table S1 for demographic and virological characteristics). The percentage of drug-naive patients harbouring drug-resistant strains is 1% (all genotype D). The only primary drug-resistance mutations detected were rtM204I (0.4%, 1/245) and rtN236T (0.4%, 1/245), while the only secondary mutations detected were rtL180M and rtV173L, each present in 0.4% of patients. Again, the presence of ≥ 1 immune-associated escape mutations in genotype D was significantly higher in drug-exposed patients with drug-resistant strains than in drug-naïve patients (29.5%[92/312] vs 21.2%[52/245], *P* = 0.032). No association was observed for genotype A (14.9%[38/255] vs 11.3% [8/71], *P* = 0.56).

**Fig. 3:**
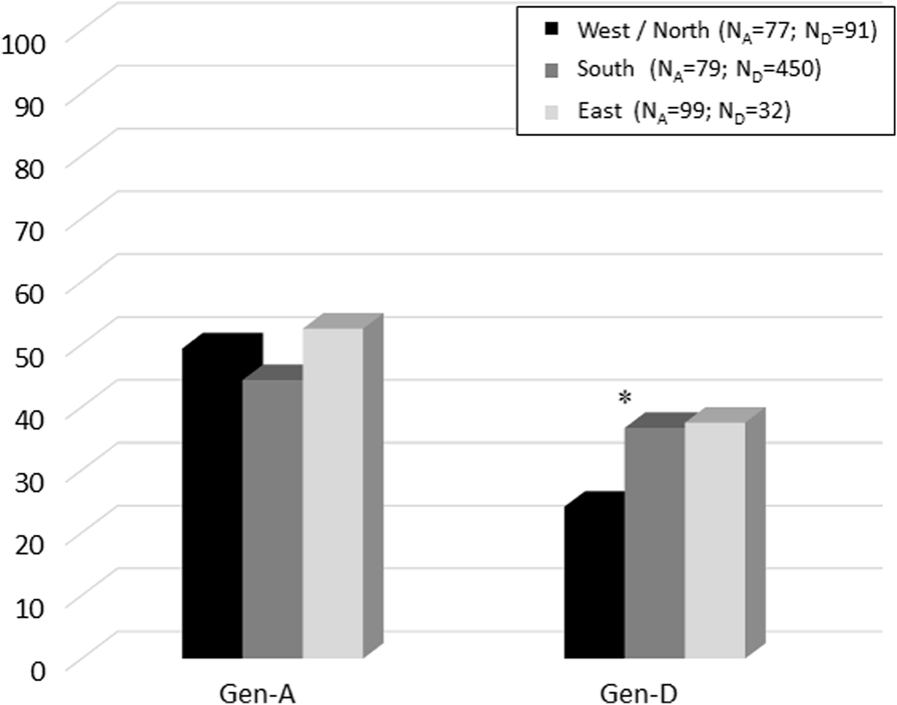
The histogram reports the percentage of patients with at least one immune-associated escape mutations between European regions. The prevalence was calculated in HBV genotype-D and -A infected patients from Western/Northern (black bars), Southern (grey bars), and Eastern Europe (light grey bars). Statistically significant differences were assessed by Chi Squared Test for independence based on a 2 × 2 contingency table. * *P* = 0.02

Our results also showed that the distribution of immune-associated escape mutations differed between European regions (Fig. 3). Indeed, the percentage of HBV genotype-D infected patients with ≥ 1 immune-associated escape mutation was significantly higher in Southern Europe than in Western/Northern Europe (36.7% vs 24.2%, *P* = 0.02). This increase was also observed in Eastern compared to Western/Northern Europe, although not statistically significant (37.5% vs 24.2%, *P* = 0.17) (Fig. 3).

By multivariable-analysis, factors independently associated with higher selection of ≥ 1 immune-associated escape mutation was genotype-D (OR[95% CI]:2.20[1.32–3.67], *P* = 0.002) and age (OR[95% CI]:1.02(1.00–1.03), *P* = 0.013) (Table 2). A trend between the presence of ≥ 1 immune-associated escape mutations and higher levels of serum HBV-DNA was also observed (OR[95% CI]:1.10[0.99–1.23], *P* = 0.079) (Table 2).

**Table 2:** Factors associated with the presence of at least one immune-associated escape mutation by fitting a uni-multivariable logistic regression model

Variables	Univariate analysis^b^		Multivariate analysis^b^	
	crude OR [95% CI]	*p*-value	adjusted OR [95% CI]	*p*-value
Gender (Female vs. Male^a^)	1.16 (0.76–1.78)	0.483	1.20 (0.77–1.87)	0.432
Age (per 1 year increase)	*1.02 (1.00–1.03)*	*0.010*	*1.02 (1.00–1.03)*	*0.013*
HBV-DNA (per 1 log_10_ IU/ml increase)	1.03 (0.94–1.14)	0.490	1.10 (0.99–1.23)	0.079
LAM	1.16 (0.65–2.08)	0.616	1.46 (0.71–3.02)	0.307
ADV	1.44 (0.96–2.17)	0.078	1.31 (0.83–2.06)	0.250
ETV^c^	1.28 (0.71–2.31)	0.409	2.04 (0.97–4.29)	0.060
TDF	0.76 (0.31–1.87)	0.547	1.13 (0.43–3.02)	0.803
Geographical origin				
South^a^	1		1	
West	0.71 (0.43–1.17)	0.175	1.03 (0.55–1.89)	0.937
North	0.55 (0.18–1.62)	0.276	0.72 (0.23–2.28)	0.581
East	0.75 (0.46–1.22)	0.250	1.26 (0.62–2.55)	0.519
Year of collection				
1997-2002^a^	1		1	
2003–2005	1.07 (0.42–2.71)	0.892	0.79 (0.28–2.20)	0.651
2006–2008	*2.37 (1.11–5.07)*	*0.026*	1.65 (0.67–4.01)	0.273
2009–2012	1.79 (0.84–3.83)	0.134	1.36 (0.50–3.68)	0.547
Genotype (D vs. A^a^)	*2.19 (1.43–3.34)*	*< 0.0001*	*2.20 (1.32–3.67)*	*0.002*

^a^ Reference group

^b^ The analysis was led on 650 patients for whom type of anti-HBV drugs received was known

^c^ Among 64 ETV-treated patients, 26 received LMV

*P*-value in italic are statistically significant

*Abbreviations*: *ADV* adefovir, *CI* Confidence interval, *ETV* entecavir, *LAM* lamivudine, *OR* Odd ratio, *TDF* tenofovir

### Detection of NA-induced immune-escape mutations

Due to RT and HBsAg open reading frames overlapping, some drug-resistance mutations in RT can correspond to some NA-induced immune-escape mutations in HBsAg. The prevalence of such mutations (sI195M, sI196S, and sE164D resulting from drug-resistance mutation rtM204 V, rtM204I, and rtV173L) was thus investigated. At least one NA-induced immune-escape mutation was detected in 28.6% (237/828) of patients (Fig. 1b). The proportion of patients with ≥ 1 drug-induced immune-escape mutation did not show statistically significant differences over time and ranged from 38.4% in 1998–2002 to 30.0% in 2009–2012.

Notably, HBV genotype-A was associated with a significantly higher prevalence of NA-induced immune-escape mutations (39.6% vs 23.7%, *P* < 0.001) (Fig. 1b). This was also confirmed by multivariable-analysis (2.03[1.32–3.10]; *P* = 0.001), along with LAM use (OR[95% CI]:4.60[1.87–11.31]; P = 0.001) (Table 3). In particular, the vaccine-escape mutational pattern sI195M + sE164D (resulting from rtM204V + rtV173L) was present in 7.1% (18/255) of HBV genotype-A infected patients and in 3.7% (21/573) of HBV genotype-D infected patients (P = 0.03) (Fig. 2b).

**Table 3:** Factors associated with the presence of at least one drug-induced immune-associated escape mutation by fitting a uni-multivariable logistic regression model

Variables	Univariate analysis		Multivariate analysis	
	crude OR [95% CI]	*p*-value	adjusted OR [95% CI]	*p*-value
Gender (Female vs. Male^a^)	0.76 (0.51–1.14)	0.188	0.71 (0.46–1.08)	0.111
Age (per 1 year increase)	1.00 (0.99–1.01)	0.672	1.00 (0.99–1.01)	0.850
HBV-DNA (per 1 log_10_ IU/ml increase)	1.06 (0.97–1.15)	0.208	1.04 (0.94–1.15)	0.409
LAM	*4.03 (1.97–8.25)*	*< 0.0001*	*4.60 (1.87–11.31)*	*0.001*
ADV	*0.42 (0.27–0.65)*	*< 0.0001*	*0.53 (0.33–0.86)*	*0.009*
ETV	0.99 (0.57–1.73)	0.981	2.02 (0.95–4.29)	0.068
TDF	1.10 (0.52–2.31)	0.805	1.58 (0.67–3.73)	0.294
Geographical origin				
South^a^	1		1	
West	1.01 (0.65–1.58)	0.951	0.75 (0.43–1.32)	0.323
North	0.58 (0.21–1.58)	0.287	0.49 (0.17–1.41)	0.184
East	*1.79 (1.18–2.71)*	*0.006*	1.22 (0.64–2.33)	0.552
Year of collection				
1997-2002^a^	1		1	
2003–2005	0.58 (0.29–1.17)	0.128	0.88 (0.41–1.92)	0.754
2006–2008	0.61 (0.34–1.08)	0.088	1.09 (0.54–2.19)	0.817
2009–2012	0.72 (0.41–1.27)	0.259	0.83 (0.36–1.88)	0.650
Genotype (A vs. D^a^)	*2.15 (1.53–3.02)*	*< 0.0001*	*2.03 (1.32–3.10)*	*0.001*

^a^ Reference group (dummy)

*P*-value in italic are statistically significant

*Abbreviations*: *ADV* adefovir, *CI* Confidence interval, *ETV* entecavir, *LAM* lamivudine, *OR* Odd ratio, *TDF* tenofovir

### Detection of stop-codons

Stop-codons determine truncated HBsAg production that can be implicated in hepatocarcinogenesis. Stop-codons were observed in 8.5% of patients (9.8%[25/255] for genotype-A vs 7.9%[45/573] for genotype-D). They occurred at 20 HBsAg-positions, including 172 (corresponding to drug-resistance mutation rtA181T) and 182, both known to increase HBV oncogenic potential (Lee et al., [38]). Notably, the selection of stop-codons at HBsAg-positions 182 and 199 occurred more frequently in genotype-A than D (4.7%[12/255] vs 1%[6/573], *P* = 0.001 and 2%[5/255] vs 0%[0/573], P = 0.001, respectively) (Fig. 2c). These results were confirmed also when the analysis was focused on LAM-treated patients (182: 4.3%[9/209] vs 1.1%[4/362], *P* = 0.013; 199: 1.9%[4/209] vs 0%[0/362], *P* = 0.008).

No associations were observed between the presence of stop-codons and the following variables: patients’ demographics, serum HBV-DNA at the time of genotypic testing, anti-HBV drugs, geographical origin, year of collection, and HBV-genotype.

## Discussion

In this largest-to-date European survey of 828 NA-experienced chronically HBV-infected patients, ≥ 1 immune-associated escape and NA-induced mutation was observed in 22.1 and 28.6% of patients, respectively. Furthermore, in 8.5% of patients, ≥ 1 stop-codon in HBsAg was detected.

The proportion of patients with ≥ 1 immune-associated escape mutation was stable to around 15% in 1998–2002 and in 2003–2005, and remained > 20% in 2006–2008 and in 2009–2012, suggesting a substantial circulation over time of viral strains with a reduced antigenic potential.

By multivariable analysis, the selection of immune-associated escape mutations (including vaccine-escape mutations) was significantly higher in HBV genotype-D than A. HBV genotype-D is known to be more prone to the onset of HBeAg-negative chronic hepatitis characterized by an extensive accumulation of mutations in the pre-core/basal core promoter of HBV-genome in response to a potent host-based selection pressure [21]. It is conceivable that this selective pressure may also favor the generation and selection of immune-associated escape mutations in HBsAg, further exacerbating HBV-escape from immunological-pressure.

Only the immune-associated escape mutation G130 N was detected more frequently in genotype-A than -D. This difference can be explained considering the fact that the number of nucleotide substitutions necessary to generate G130 N from the wild-type amino acid is lower in genotype-A than -D [22]. This suggests that the different genetic background of HBV-genotypes can modulate the generation of immune-associated escape mutations, and consequently HBV-antigenicity.

Recent studies highlighted the role of immune-associated escape mutations in immunosuppression-driven HBV-reactivation [6–9, 23]. It has been proposed that immune-associated escape mutations can favor the re-uptake of HBV-replication during the initial weakening of immune-system, particularly during rituximab-treatment (known to deplete B-lymphocytes) [6]. The substantial circulation of immune-associated escape mutations may thus pose an issue in term of increased risk of HBV-reactivation in immunosuppressed-patients.

Previous in-vitro studies showed that some immune-associated escape mutations can promote the fitness of HBV lamivudine-resistant strains [23, 24]. We found an enrichment of immune-associated escape mutations in drug-exposed patients with drug-resistant strains compared to drug-exposed patients with wild-type virus and to drug-naïve patients. This highlights a strict relationship between drug-resistance and immune-associated escape mutations, and suggests the ability of immune-associated escape mutations to stabilize drug-resistance mutations in viral-quasispecies. We observed that sP120T significantly correlated with rtM204V/I, and their co-presence is characterized by elevated serum HBV-DNA. This is consistent with an in-vitro study showing sP120T ability to rescue HBV-replication impaired by rtM204V/I [24].

The ability of immune-associated escape mutations to promote the fitness of HBV lamivudine-resistant strains can raise the issue on lamivudine-use as prophylaxis in immunosuppressed-patients, and highlights the importance to use potent anti-HBV drugs in order to prevent HBV-reactivation. Since the highly potent anti-HBV drugs will soon become generic, this will also allow to reduce the cost related to the management of immunosuppressed-patients at risk of HBV-reactivation.

This has also implications for those European Countries in which lamivudine is still prescribed, again supporting the role of potent anti-HBV drugs for a proper management of patients with chronic HBV-infection.

The circulation of immune-associated escape mutations can have important implications, since they can potentially affect the efficacy of the current vaccination strategy. Indeed, several studies have highlighted the presence of immune-associated escape mutations in individuals who contracted HBV-infection despite completed HBV-vaccination [25–27]. In a study led in Taiwan, a positive HBV-DNA was detected in 10 of 60 individuals in which the HBsAg or anti-hepatitis B core (HBc) was either positive or equivocal despite vaccination [27]. Among them, 8 have received 3 doses of vaccine. Five out of 8 vaccinees harbored HBsAg mutations: 4 with immune-associated escape mutations, and 1 with a stop-codon in HBsAg [27].

Immune-escape mutations can also play a relevant role in the setting of mother-to-child transmission. Currently, HBV-vaccine (in addition to immunoglobulins) is administered to children born to HBV-infected mothers. In a recent study, serum HBV-DNA was detected in 28% children born from HBsAg-positive mothers, and fully responded to HBV-vaccination. Among them, 62% infected children had ≥ 1 immune-associated escape mutation, suggesting the maternal transmission of viral strains with enhanced capability to evade neutralizing antibodies in vaccinated-children [28].

In chronic HBV-infection, recent studies highlighted that the presence of immune-associated escape mutations at baseline was negatively correlated with HBsAg-loss during treatment with potent anti-HBV drugs [29, 30]. It is conceivable that the circulation of these mutations can hamper the full immune control of the virus despite potent anti-HBV therapy. This issue should be considered by the recent therapeutic strategies aimed at achieving HBV-cure.

Finally, different studies showed that some immune-associated escape mutations can affect HBsAg-quantification by altering HBsAg-binding to antibodies used in diagnostic assays [6, 31, 32]. HBsAg-amount is used to provide a more precise definition of the inactive carrier status and to monitor the efficacy of interferon-treatment. The presence of immune-associated escape mutations may cause an underestimation of HBsAg-levels thus hampering the proper management of chronically HBV-infected patients.

Due to the peculiar HBV-genome organization, drug-resistance mutations rtM204 V, rtM204I, and rtV173 L correspond to the NA-induced immune-escape mutations sI195M, sI196S, and sE164D. In our study, HBV genotype-A was associated with a significantly higher prevalence of NA-induced immune-escape mutations. This is in line with previous studies showing that genotype-A is more prone to develop rtM204V than genotype-D at lamivudine failure [32–34]. The issue of NA-induced escape mutations is critical considering the ongoing use of lamivudine in some European regions where genotype-A is predominant [18, 35].

Finally, ≥ 1 stop-codon was detected in 8.5% of patients. Stop-codons can determine the accumulation of truncated HBsAg in the endoplasmic-reticulum, thus inducing oxidative stress and in turn enhancing hepatocytes proliferation [36, 37]. They were detected at 20 HBsAg-positions including 172 and 182, known to promote the carcinogenic transformation of hepatocytes [38, 39]. Notably, stop-codon at HBsAg-position 172 derives from the drug-resistance mutation rtA181T selected under ADV- and (in some cases) LAM-treatment [39]. This represents an important issue probably originating from the broad use of first-generation drugs which may have fuelled the circulation of viral strains with an increased oncogenic potential.

## Conclusions

“Immune-escape mutations and stop-codons develop in a large proportion of NA-exposed patients in Europe. These mutant isolates may potentially transmit in general population, including vaccinated individuals, and fuel drug-resistance emergence”.

## Additional file

Additional file 1: **Table S1.** Demographic and virological characteristics of HBV genotype-D drug-naïve patients. (DOCX 12 kb)

## Abbreviations

ADV: Adefovir dipivoxil
ALT: Alanine aminotransferase
CHB: HBV-infected patients
ESAR: European Society for translational antiviral research
ETV: Entecavir
HBc: Hepatitis B core
HbeAg: Hepatitis B e antigen
HbsAg: HBV surface antigen
HBV: Hepatitis B virus
HCC: Hepatocellular carcinoma
IQR: Interquartile range
LAM: Lamivudine
LdT: telbivudine
NAs: Nucleos(t)ide analogues
OR: Odds ratio
RT: Reverse transcriptase
TAF: Tenofovir-alafenamide
TDF: Tenofovir
