# Supplementary material for: Immune-escape mutations and stop-codons in HBsAg develop in a large proportion of patients with chronic HBV infection exposed to anti-HBV drugs in Europe
Source: BMC Infect Dis. 2018 Jun 1;18:251. doi: 10.1186/s12879-018-3161-2 (PMC5984771; doi:10.1186/s12879-018-3161-2)
Supplement: Supplementary file 1 — Table S1. Demographic and virological characteristics of HBV genotype-D drug-naïve patients. (DOCX 12 kb) [file 12879_2018_3161_MOESM1_ESM.docx]

**Table S1. Characteristics of HBV genotype-D drug-naïve patients**

|  | **Overall (N=254)** | **Datum avaibility** |
| --- | --- | --- |
| **General** |  |  |
| Median Age (IQR), years | 48 (36-59) | 200/254 |
| Male, N(%) | 153 (64) | 239/254 |
| **HBV-status** |  |  |
| Median HBV-DNA, log IU/ml (IQR) | 3.5 (2.7-4.8) | 221/254 |
| HBeAg positive, N(%) | 19 (9.9) | 192/254 |
| HBsAg positive, N(%) | 179 (97.3) | 184/254 |
| Median ALT, IU/L (IQR) | 35 (26.3-62.7) | 184/254 |
| Median AST, IU/L (IQR) | 28 (18-50) | 182/254 |
